# Apoptotic gene expression in melanoma cells treated with kaurenic acid

**DOI:** 10.1186/1753-6561-6-S6-P45

**Published:** 2012-10-01

**Authors:** María B Rivero, Hernán J Mendoza, Miguel AChiurillo, Miriam C Sosa-Seguera

**Affiliations:** 1 Laboratorio de Genética Molecular, Yunis-Turbay Departamento de Ciencias Funcionales Medicina, Universidad Centrooccidental Lisandro Alvarado, Venezuela; 2 Unidad de Farmacoiogía Experimental, Departamento de Ciencias Funcionales Medicina, Universidad Centrooccidental Lisandro Alvarado, Venezuela

## 

Terpenoids have been described as pharmacologically active substances. Antibacterial, antifungal, anti-inflammatory activities and cytotoxicity against cancer cell lines are some of the biological effects described for these compounds [1-4]. Kaurenic acid is a diterpene isolated from the aerial parts of *Espeletia semiglobulata* (Compositae) and its antitumor effect via apoptosis and necrosis against melanoma cells in animal models has been described [5]. The present study researches the molecular mechanism for this anti-melanoma effect on cells inoculated in mice. One hundred thousand cells of melanoma B16F1 were used for inoculation in fifteen male, 20 g weight C57BL/6 mice. There were three groups of five mice each: group A was treated for 21 days with saline solution (0.009%/day); group B was treated for 21 days with Taxol (14.5 mg/kg/week); and group C was treated with kaurenic acid for 21 days (1 mg/kg/day). Two animals in group C died during the treatment. The expression of genes for proteins Bcl-2, Bax-α, Bcl-xL, c-Myc, P53, ICE (caspase 1), ICH1 (caspase 2), CPP32 (caspase 3), Apa F1 (activator), TNF-α, eNOS, Il1-β, iNOS, nNOS, Flice (caspase 8), MCH6 (caspase 9) involved in different apoptotic pathways, was qualitatively assessed by using Multiplex PCR Maxim Biotech Inc. Kits for Mouse Apoptotic Genes, with GAPDH and 18S as internal controls in cDNA synthesized by Trizol method [6]. Each PCR was repeated twice in the same conditions for each sample. Amplification products were visualized in agarose gels cored with ethidium bromide and illuminated with ultraviolet light. The results showed that there was no expression of *Bcl-xL* in any of the animals from groups B and C. Furthermore, there was no expression of *iNOS*, *nNOS* and *eNOS* in samples from group C. Other evaluated genes were present in all groups. The alteration in the expression of the *Bcl-xL* gene (anti-apoptotic protein) and nitric oxide family proteins could be crucial events for the anti-melanoma effect. Kaurenic acid could offer potential usefulness as an agent for therapy of this cancer.
